# Comparative metagenomics of *Daphnia *symbionts

**DOI:** 10.1186/1471-2164-10-172

**Published:** 2009-04-21

**Authors:** Weihong Qi, Guang Nong, James F Preston, Frida Ben-Ami, Dieter Ebert

**Affiliations:** 1 Swiss Tropical Institute, Socinstrasse 57, 4002 Basel, Switzerland; 2 Department of Microbiology and Cell Sciences, University of Florida, Gainesvillle, FL 32611, USA; 3 Zoological Institute, Basel University, Vesalgasse 1, 4051 Basel, Switzerland; 4 Functional Genomics Center Zurich, UNI/ETH Zurich, Winterthurerstrasse 190, 8057 Zurich, Switzerland

## Abstract

**Background:**

Shotgun sequences of DNA extracts from whole organisms allow a comprehensive assessment of possible symbionts. The current project makes use of four shotgun datasets from three species of the planktonic freshwater crustaceans *Daphnia*: one dataset from clones of *D. pulex *and *D. pulicaria *and two datasets from one clone of *D. magna*. We analyzed these datasets with three aims: First, we search for bacterial symbionts, which are present in all three species. Second, we search for evidence for Cyanobacteria and plastids, which had been suggested to occur as symbionts in a related *Daphnia *species. Third, we compare the metacommunities revealed by two different 454 pyrosequencing methods (GS 20 and GS FLX).

**Results:**

In all datasets we found evidence for a large number of bacteria belonging to diverse taxa. The vast majority of these were Proteobacteria. Of those, most sequences were assigned to different genera of the Betaproteobacteria family Comamonadaceae. Other taxa represented in all datasets included the genera *Flavobacterium, Rhodobacter, Chromobacterium, Methylibium, Bordetella, Burkholderia *and *Cupriavidus*. A few taxa matched sequences only from the *D. pulex *and the *D. pulicaria *datasets: *Aeromonas, Pseudomonas *and *Delftia*. Taxa with many hits specific to a single dataset were rare. For most of the identified taxa earlier studies reported the finding of related taxa in aquatic environmental samples. We found no clear evidence for the presence of symbiotic Cyanobacteria or plastids. The apparent similarity of the symbiont communities of the three *Daphnia *species breaks down on a species and strain level. Communities have a similar composition at a higher taxonomic level, but the actual sequences found are divergent. The two *Daphnia magna *datasets obtained from two different pyrosequencing platforms revealed rather similar results.

**Conclusion:**

Three clones from three species of the genus *Daphnia *were found to harbor a rich community of symbionts. These communities are similar at the genus and higher taxonomic level, but are composed of different species. The similarity of these three symbiont communities hints that some of these associations may be stable in the long-term.

## Background

Metagenomics is the field that infers the properties of a habitat through the analysis of genomic sequence information obtained from a sample usually collected from a single habitat. The sequences are usually compared to databases, with the aim to characterize the biological community of this habitat. Among the advantages of this explorative method are the free and uncomplicated sampling of the material, the possibility of obtaining sequences from unknown and unculturable organisms, the absence of any taxonomic restrictions and the relative ease of conducting such studies [1-4]. Metagenomics studies have been done in various habitats, including sea water [5], ice cores [6] and deep mine communities [7]. Of particular recent interest has been the application of metagenomic approaches to study samples obtained from organisms, which harbor various symbionts, such as unknown and uncultuable bacteria, protozoa or viruses. For example, the symbiont communities of honey bees [8], the guts of mice [9] and humans [10], marine sponges [11], oligochaetes [12] and plant-rhizobacteria [13] revealed many new symbiont taxa. However, not only samples collected with the aim to find symbionts revealed previously unknown organisms, but also datasets from genome projects where one single genome was targeted may contain sequences of other species, presumably symbionts [14]. Here we report on the bacterial communities associated with three clones each from one species of crustaceans of the genus *Daphnia*, which had been used in genome projects and revealed besides sequences to the targeted species, a rich body of sequences to other species. We use the term symbiont to include organisms that were found to be associated with the samples of these *Daphnia*, disregarding whether they are parasites, commensals or mutualists. We cannot rule out, that some of these organisms are independent of the *Daphnia*, e.g. free living bacteria in the water, parts of the ingested food or contaminants from handling the samples. For simplicity we use the term symbiont throughout this article.

**Figure 1:**
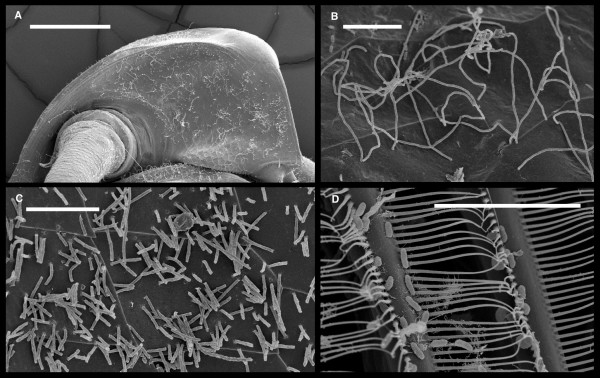
**Four examples of scanning electron microscopic (SEM) images of parts of *D. magna *showing numerous bacteria attached to different surface structures**. A. Head of *D. magna*. The white filamentous structures on the surface are bacteria. B and C. Surface of the carapace with bacteria attached. The thin lines on the carapace denote epidermis cell boundaries. D. Parts of the filter apparatus of *D. magna*. The oval objects are bacteria. None of the bacteria have yet been identified. Scale bar 200 μm in A and 10 μm in B, C and D.

*Daphnia *is a genus of small freshwater plankton living in standing freshwater bodies. Their body sizes ranges from 0.3 to 5 mm. They are primary consumers in the aquatic food chain and their ecology and evolution has been intensively studied [15]. Numerous ecto- and endo-parasites have been described [16,17], but the non-parasitic bacterial symbionts of *Daphnia *are very poorly known. Electron micrographs typically reveal large numbers of bacteria associated with *Daphnia*, as is illustrated with the examples in Figure 1. The entire body of *Daphnia *can be coated in thick bacterial mats [16,17]. Thus, *Daphnia *are likely to carry a community of prokaryotes with them. Only one case of a possible mutualist has been reported so far. Chang and Jenkins [18] reported the presence of photosynthetically active gut endosymbionts in *Daphnia obtusa*. They speculate that the *Daphnia *take up plastids via phagocytosis, after the lysis of the mother cell in the gut. Variations in ultrastructure lead them to assume that plastids from different sources are taken up, including Cyanobacteria. These findings have not been confirmed for any other *Daphnia *species, although the ecological niches of *Daphnia *species are often strongly overlapping.

Here we take advantage of shotgun sequences obtained from three laboratory clones (= iso-female lines) each from one *Daphnia *species to search for indications of bacterial and plastid symbionts. For this we compared the sequences against the NCBI-nt database on nucleotide sequences using BLASTN [19] and analyzed and ordered the results using the metagenomics software MEGAN [20]. This software allows the exploration of the taxonomic content of a community sample based on the NCBI taxonomy. Community shotgun datasets represent sequences independently sampled from random regions of genomes randomly selected from a given community. These sequences can have very different levels of conservation. Without any assumptions about the functions of the sequences used, MEGAN associates each sequence to the lowest common ancestor of the set of taxa it hits. Thus, species specific sequences are assigned to low order taxa such as species or strains, while widely conserved sequences are assigned to high-order taxa. In other words, the taxonomical level of the assigned taxon reflects the level of conservation of the sequence. The strength of this statistical approach is that it makes use of all kind of sequences for taxon identification. Therefore, when using random sequences MEGAN, will usually show better taxonomic resolution than an analysis using only a small set of phylogenetic markers [20]. This type of analysis is in particular useful when, as is the case here, datasets are analyzed, which were obtained by random shotgun sequencing, rather than targeted sequencing (see also [21]) and where the length of the sequence reads are short [20,22].

Our choice to use the software MEGAN for the analysis of the datasets from the *Daphnia *projects is based on several aspects, which help to reduce known problems in comparative metagenomics. A known shortcoming of the assignment of sequences to taxonomic groups is its inability to deal with horizontally transferred genes and the inability of mapping sequences to internal nodes of the tree [23]. However, these problems are mainly of concern when using "best-BLAST-hit" mapping. The software MEGAN was developed to avoid this problem (see previous paragraph). A further problem of assigning sequences to taxonomic groups is the well know bias in the taxon representation in our databases [24,25]. This problem cannot be fully solved, but the ability of MEGAN to assign sequence to the lowest common ancestor, ameliorates the consequences of a database bias. Sequences will be assigned to the common ancestor of the true species in question and those being represented in the database. Novel sequences will not be assigned at all [20].

The aims of our analysis were first to compare the shotgun sequences of the prokaryote communities coming from three *Daphnia *species. Second to test if the shotgun sequences give evidence for a plastid symbiont in *Daphnia *as had been suggested [18]. Third, to estimate the repeatability of a metagenomics approach using two different sequencing platforms, the pyrosequencers GS 20 and GS FLX [26] for one of the three *Daphnia *species.

## Results and discussion

In the four datasets, sequences that were assigned to known cellular organisms varied from 9% to 18% (Table 1). The vast majority of the assigned sequences were to Eukaryota and to Bacteria. Few sequences were assigned to the NCBI Taxonomy categories: Archaea, Viroids, Other and Unclassified. Only among the *D. pulicaria *sequences were hits (a total of 4) found to viruses. However, the low bit scores suggest that these may have other origins. As the scaffolds of *D. pulex *included in this study had been presorted to include only bacteria, there might have been more hits to taxa other than Bacteria and Eukaryota.

**Table 1:** Number of sequences assigned and unassigned in the MEGAN analysis.

*Daphnia *species/dataset	Assigned to cellular organisms	Assigned to Bacteria without Firmicutes^1^	Not assigned^2^	Sequences without hits
*D. pulex*	38,249	25,868	97,852	120,355
*D. pulicaria*	99,178	25,604	966,027	23,469
*D. magna *GS 20	3,028	2,560	16,007	26
*D. magna *GS FLX	4,781	4,285	21,535	12

^1 ^The Firmicutes were excluded, because the *D. magna *datasets contained a bacterial parasite belonging into this taxon. For each dataset, the sum of columns 2, 4, and 5 is less than the total number of sequences analyzed (Table 2) due to the few sequences assigned to other NCBI taxonomy categories such as "Other" and "Unclassified".

^2 ^The unassigned sequences are sequences without hits above the defined thresholds (See Materials and Methods). They may be A) sequences that do not have homologs in the current NCBI-nt database, B) sequences that evolved so strongly that their homologs are disguised by bit scores below our threshold or C) sequences that are assigned to species to which no other sequences is assigned (min-support threshold = 2).

**Figure 2:**
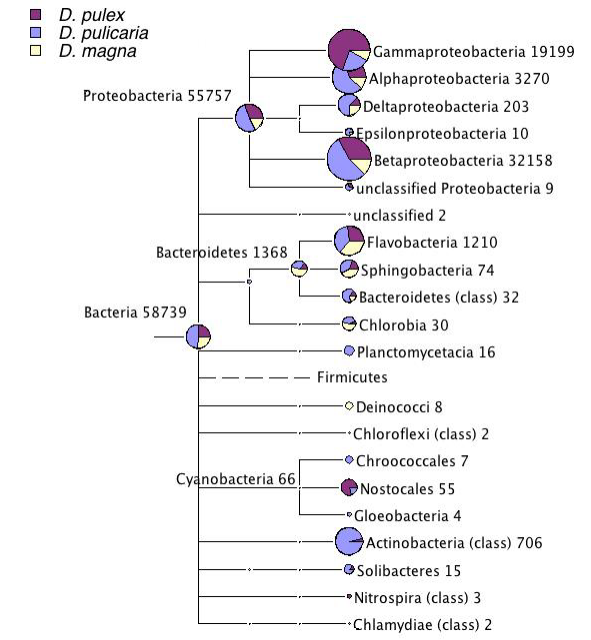
**The comparative taxonomic tree of the bacterial orders found in the three *Daphnia *datasets**. The data of the two *D. magna *datasets were combined for this figure. Only bacterial orders, with at least 2 sequences assigned are included. The Firmicutes were excluded (see text for explanation). The numbers next to the taxon names are the cumulative number of sequences assigned to this taxon. The size of the circles is proportional to the number of sequences assigned to this node. The color scheme of each pie chart is as the following: dark dull magenta for *D. pulex *sequences, pale dull blue for *D. pulicaria *sequences, vanilla for *D. magna *sequences.

The numbers of bacterial genera (excluding the Firmicutes) with at least two reads assigned were 90, 123, 37 and 51 for the *D. pulex, D. pulicaria, D. magna *GS 20 and *D. magna *GS FLX datasets, respectively. The lower number of genera revealed by the *D. magna *datasets corresponds with the smaller size of these datasets (Table 1, Table 2). This large number of genera indicates a rich community of bacteria in and on *Daphnia*. In all datasets the majority of the sequences were assigned to the Gamma- and Betaproteobacteria (Fig. 2), which together accounted for more than 87% of the sequences assigned to bacteria. Outside the Proteobacteria, the Bacteroidetes and to a lesser degree to the Actinobacteria were found, the later however, mainly in the *D. pulicaria *dataset. Except the Actinobacteria, all taxa with substantial number of sequences assigned to were found in datasets from all three *Daphnia *species.

**Table 2:** Summary of the four datasets included in this analysis.

	*D. pulex*	*D. pulicaria*	*D. magna *GS 20	*D. magna *GS FLX
**Original input data:**				

Data type	Possible bacterial scaffolds	Contigs and raw reads longer than 500 bps	Contigs longer than 100 bps	Contigs longer than 100 bps

No. of original sequences	21,646	327,632	4,388	6,696

Total length (bps)	59,379,440	323,393,910	4,335,734	6,154,579

Average length (mean ± stdev bps)	2,743 ± 7,205	987 ± 255	988 ± 2,830	919 ± 2,507

Median length (bps)	975	993	218	280

Minimum length (bps)	10	500	100	100

Maximum length (bps)	216,125	9,681	40,374	40,088

**Sequence fragments subjected to BLASTN:**				

No. fragments	256,498	1,088,697	19,163	26,430

Total length (bps)	133,734,869	570,776,073	8,809,340	12,259,583

Average length (mean ± stdev bps)	521 ± 100	524 ± 195	459 ± 149	463 ± 131

Median length (bps)	500	500	500	500

### Assignment of sequences to the bacteria, without Firmicutes and Cyanobacteria

**Figure 3:**
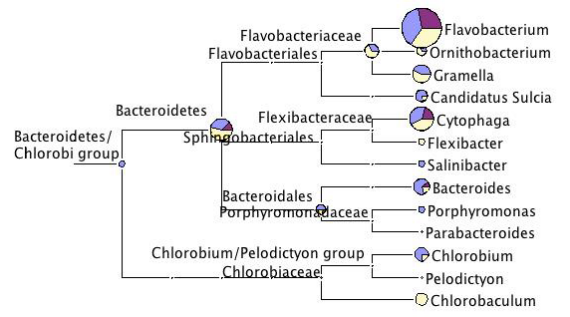
**Taxonomic diversity of the three *Daphnia *datasets within the Bacteroidetes/Chlorobi group**. For more explanation see legend to Fig. 2.

The majority of the assigned sequences fall on two phyla, the Bacteroidetes and the Proteobacteria. Among the Bacteroidetes, most sequences were assigned to the Flavobateriales (between 187 to 463 sequences per sets, or 1.3 – 7.7% of the sequences) and a very large proportion of those to the genus *Flavobacterium *(Fig. 3). Within this genus, no single species stuck out as giving a better match than other species. *Flavobacteria *are a group of opportunistic pathogens (e.g. salmon), commensals (e.g. in infusoria, cnidaria) [27] and intracellular symbionts of insects [28-30]. They are widely distributed in freshwater habitats, but also occur in association with terrestrial hosts. Some members of *Flavobacteria *are known to play a significant role in the degradation of proteins, polysaccharides, and diatom debris in natural environments [31,32]. Cultured representatives of Flavobacteria with ability to degrade various biopolymers such as cellulose, chitin and pectin were described [33]. The commonness in all datasets here indicates that they may indeed be symbionts of *Daphnia*. One may speculate that *Flavobacterium *may play a role in food digestion in *Daphnia*, which mainly feed on unicellular planktonic algae [34]. This hypothesis has to be tested with a targeted approach.

Another genus of the Bacteroidetes, which was consistently found in all datasets is *Cytophaga *(Fig. 3) These are gliding bacteria found in freshwater and marine habitats, in soil and in decomposing organic matter. However, hits to this genus were never frequent (between 10 and 25 hits).

The phylum Proteobacteria attracted 98, 94, 84 and 88% of the sequences assigned to bacteria in the *D. pulex, D. pulicaria, D. magna *GS 20 and *D. magna *GS FLX datasets, respectively. Table 3 shows the distribution of all Proteobacteria genera for which at least one dataset attracted more than 1% of the sequences assigned to Bacteria.

**Table 3:** Taxa within the Proteobacteria, which attracted at least 1% of the sequences within at least one of the four datasets.

Taxon level	Taxon	*D. pulex*	*D. pulicaria*	*D. magna *GS 20	*D. magna *GS FLX	Average
Class	**Alphaproteobacteria**	3.9	8.0	4.5	6.6	5.7

Genus	*Rhodobacter*	0.4	1.4	2.0	2.8	1.6

Class	**Betaproteobacteria**	41.9	72.7	63.0	63.5	60.3

Family	Neisseriaceae	0.1	1.2	0.0	0.3	0.4

Genus	*Chromobacterium*	0.1	1.2	0.0	0.2	0.4

Order	Burkholderiales	41.0	69.5	61.8	61.8	58.5

Genus	*Methylibium*	2.8	3.9	1.0	1.2	2.2

Family	Alcaligenaceae	0.3	0.6	1.2	1.1	0.8

Genus	*Bordetella*	0.3	0.4	1.2	1.1	0.7

Family	Burkholderiaceae	1.3	3.0	2.2	1.9	2.1

Genus	*Burkholderia*	0.3	1.1	0.3	0.3	0.5

Genus	*Cupriavidus*	0.5	1.0	1.4	1.1	1.0

Family	Comamonadaceae	32.0	56.5	53.0	53.1	48.7

Genus	*Acidovorax*	9.9	10.5	16.0	16.3	13.2

Genus	*Rhodoverax*	0.9	4.1	3.2	2.8	2.8

Genus	*Polaromonas*	3.9	12.8	14.6	14.7	11.5

Genus	*Delftia*	2.5	5.5	0.2	0.2	2.1

Genus	*Verminephrobacter*	6.8	4.8	4.4	4.6	5.2

Class	**Gammaproteobacteria**	53.0	16.8	29.6	27.2	31.6

Genus	*Pseudomonas*	43.3	11.5	0.8	1.5	14.3

Genus	*Serratia*	8.6	0.0	0.0	0.0	2.2

Genus	*Aeromonas*	0.1	3.8	0.0	0.0	1.0

Genus	*Escherichia*	0.1	0.0	4.6	4.4	2.3

Cell entries are percentages of the number of sequences assigned to the Proteobacteria.

**Figure 4:**
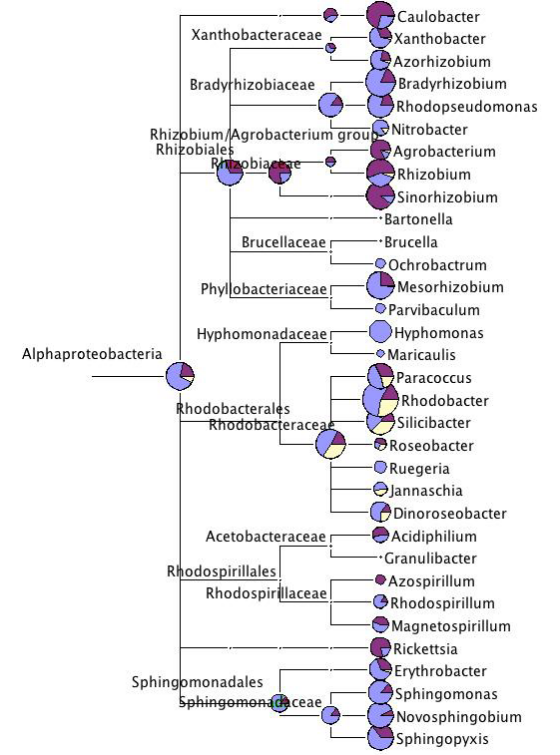
**Taxonomic diversity of the three *Daphnia *datasets within Alphaproteobacteria**. For more explanation see legend to Fig. 2.

The Alphaproteobacteria attracted a lager number of hits (3.9 to 8% of sequences), with the genus *Rhodobacter *being the most common in all three *Daphnia *species (0.4 to 2.8% of reads) (Fig. 4). Other genera of the Alphaproteobacteria were only found in the *D. pulex *or the *D. pulicaria *datasets (Fig. 4). Alphaproteobacteria are commonly found in freshwater environments, including sewage. They are known for a wide range of metabolic capabilities. *Rhodobacter *were isolated from sea and freshwater.

**Figure 5:**
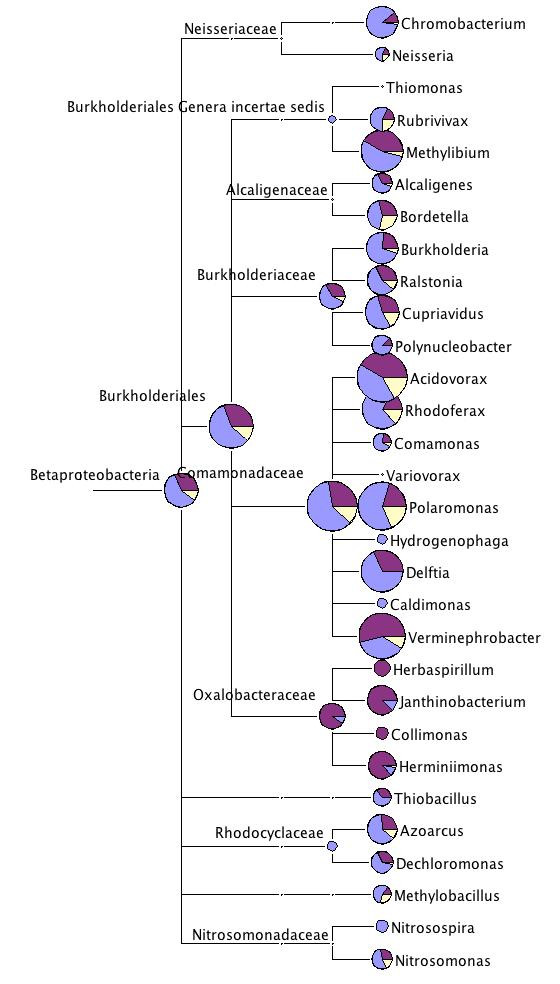
**Taxonomic diversity of the three *Daphnia *datasets within Betaproteobacteria**. For more explanation see legend to Fig. 2.

The majority of the sequences assigned to the Proteobacteria (overall about 50% of sequences) where assigned to the Burkholderiales within the Betaproteobacteria (Fig. 2, Table 3). Within the Burkholderiales, one family, the Comamonadaceae accounted for most of these hits (Fig. 5). The Comamonadaceae is a family of gram-negative aerobic bacteria, encompassing the acidovorans rRNA complex. Some species are pathogenic for plants. Within this family four genera (*Acidovorax, Rhodoverax, Polaromonas *and *Verminephrobacter*) showed up repeatedly and in high numbers in all datasets (Table 3, Fig. 5). The genera *Acidovorax *and *Polaromonas *were particularly common. A further genus, *Delftia *was only common in the *D. pulex *and *D. pulicaria *sequences (Fig. 5).

A few other genera within the Betaproteobacteria attracted relatively high numbers of sequences across all or most of the datasets: *Chromobacterium, Methylibium, Bordetella, Burkholderia *and *Cupriavidus *(Table 3, Fig. 5). Of those *Methylibium petroleiphilum *was highly represented. However, a closer inspection of the sequence alignments indicates that the species in our datasets is not exactly this, but a related species.

**Figure 6:**
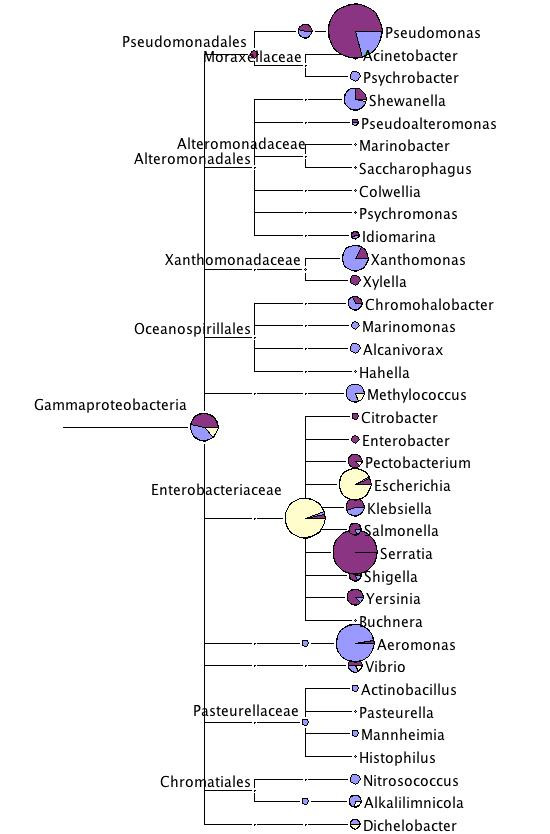
**Taxonomic diversity of the three *Daphnia *datasets within Gammaproteobacteria**. For more explanation see legend to Fig. 2.

Four genera within the Gammaproteobacteria attracted larger numbers of sequences, but in contrast to the genera in the other classes of the Proteobacteria, here the distribution was not even across the datasets (Table 3, Fig. 6).

Hits to species of the genus *Aeromonas *were found in large number in the *D. pulicaria *dataset, but hardly in the other sets (Table 3, Fig. 6). Hits were mainly to *A. hydrophila *and *A. salmonicida*, but similarities were below 100%. Both can live under aerobic or anaerobic conditions and are found in water. *A. hydrophila *is an opportunistic pathogen of humans, *A. salmonicida *causes the fish disease, furunculosis.

The single most often assigned genus in the entire analysis was *Pseudomonas *in the *D. pulex *dataset (10,994 assigned reads, 43.3%). These hits were mainly to the species *P. fluorescens *(7,067 reads), and in particularly to the strain PfO-1. Similar, but not as extreme was the presence of the same bacterium in the *D. pulicaria *sequences (Table 3, Fig. 6). The *P. fluorescens *PfO-1 genome project was run in the same genome center (The DOE Joint Genome Institute (JGI, ) where the *D. pulex *and the *D. pulicaria *sequences were obtained and it seemed possible, that these hits reflect a contamination in the *D. pulex *scaffolds, rather than a symbiont of *D. pulex*. However, inspection of bit scores and sequence identity values in the BLASTN outputs indicated that the *Daphnia *symbiont is clearly not *P. fluorescens *PfO-1. The *P. fluorescens *group includes diverse bacteria that are found in soil, but also in aquatic environments.

A further contamination candidate is the Gammmaproteobacterium *Serratia*, to which we found 2,184 matched sequences in the *D. pulex *genome. However, it is hardly seen among the *D. pulicaria *sequences, and not seen at all among the *D. magna *sequences (Table 3, Fig. 6). The species to which most sequences were assigned is *Serratia proteamaculans *568, whose genome was sequenced as well by the DOE Joint Genome Institute. Also here, the inspection of the BLASTN results indicated high similarity, but few perfect matches, excluding contamination at the JGI. *Serratia *are often associated with the human gut, but are not pathogenic.

Another genus with many hits to the *D. pulex *and the *D. pulicaria *sequences, but not to the *D. magna *sequences (Table 3), is the already mentioned Betaproteobacterium *Delftia *(Fig. 5). The DOE Joint Genome Institute sequenced *Delftia acidovorans *strain SPH-1, which is the strain most of the sequences were assigned to. However, inspection of the BLASTN results again showed that the *Daphnia *symbiont is clearly not *D. acidovorans *strain SPH-1.

**Figure 7:**
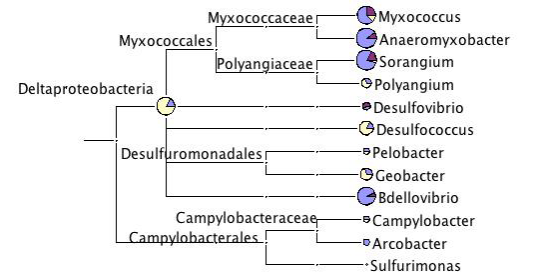
**Taxonomic diversity of the three *Daphnia *datasets within Delta- and Epsilonproteobacteria**. For more explanation see legend to Fig. 2.

About 200 sequences matched Deltaproteobacteria (Fig. 2). Within this order various taxa matched sequences from the datasets. However, there was no consistent picture across the three *Daphnia *species (Fig. 7).

### Searching for Cyanobacteria and plastid sequences

**Figure 8:**
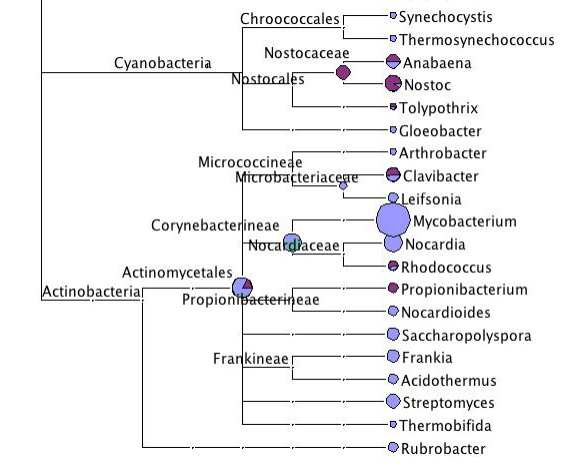
**Taxonomic diversity of the three *Daphnia *datasets within Cyanobacteria and Actinobacteria**. For more explanation see legend to Fig. 2.

Following the suggestion of Chang and Jenkins [18] that *Daphnia *may carry symbiontic plastids or cyanobacteria with them, we looked more closely into these two groups. The *D. magna *sequences revealed no hit to any Cyanobacteria taxon. Of the *D. pulex *sequences 44 (= 0.17% of the assigned sequences) were assigned to the Nostocales, a taxon of the Cyanobacteria. 19 (= 0.074%) of these hits were to the genus *Nostoc*. In the *D. pulicaria *we found 22 sequences assigned to the Cyanobacteria, half of which were to the Nostocales (Fig. 8).

The *D. pulicaria *dataset revealed 23 sequences assigned to plastids. One of them was a short sequence (100 bps) to the chloroplasts of the green algae *Chlamydomonas*, the other to the chloroplasts of flowering plants. Hits to the later came mostly from one scaffold and had high bit scores (> 500) and similarities of more than 90%. The *D. pulex *sequences revealed no hits to plastids, but this is not surprising, as the dataset had been sorted out to contain predominately prokaryote sequences. The *D. magna *GS 20 dataset did not reveal any hits to plastids. The *D. magna *GS FLX sequences contained a short sequence (104 bps) matched to a plastid, the chloroplast of the green algae *Stigeoclonium helveticum*.

The presence of plastid sequences in *Daphnia *shotgun datasets has however, to be looked at with care, as unicellular green algae are the main food of *Daphnia*, both in the field and in the laboratory [34,35]. However, the few sequences assigned to plastids here seem not to correspond closely with the algae, which were used to feed the *Daphnia *in the cultures, before they were used for DNA extraction. The *D. magna *and the *D. pulex *clone had been kept on an exclusive diet of the green algae *Scenedesmus *sp. and the *D. pulicaria *clone on a diet of the green algae *Ankistrodesmus falcatus*.

All in all we consider this as rather weak evidence for plastid symbionts in these *Daphnia *samples. The original finding was done in *D. obtusa *[18], which was not included in our study. The authors had observed variation in the type and frequency of plastid occurrence in this species, so it may not be surprising that things are different in other species. Furthermore, the long maintenance of the *Daphnia *clones in laboratory cultures may have contributed to a loss of plastids. Therefore, the absence of evidence from our metagenomics analysis is certainly not evidence for the absence of possible plastid symbionts in *Daphnia*.

### Searching for 16S rDNA sequences

All four datasets were also analyzed with a more conventional approach, which was to identify contigs/scaffolds similar to known 16S rDNA sequences. We compared our data with a collection of 471,792 16S rDNA sequences collected by the Ribosomal Database Project (RDP release 9 update 57) [36]. In total, 27 16S rDNA fragments were identified in the *D. pulicaria *dataset, 13 in the *D. pulex*, 14 in the *D. magna *GS 20, and 11 in the *D. magna *GS FLX. Of those, 17, 11, 9, and 10 bacterial species could be inferred in the *D. pulicaria*, *D. pulex*, *D. magna *GS 20, and *D. magna *GS FLX dataset, respectively. Other partial 16S rDNA sequences were identical or almost identical to regions conserved across species, thus could not be used to infer the species. In Table 4 we listed close to full length 16S rDNA sequences found in the four datasets. The nucleotide sequence identity between these sequences and their corresponding best matches ranged from 91% to 100%. Most best matched 16S rDNAs to our sequences were from uncultured bacteria. Bacterial species that could be inferred using 97% sequence identity as the cutoff value included *Pseudomonas *sp., *E. coli*/*Shigella *and the already discussed (see above) *Flavobacterium *sp. (Table 4). In both *D. pulex *and *D. pulicaria *datasets, sequences highly similar to 16S rDNA of unclassified aquatic bacterium R1-B19 were found, an undescribed beta proteobacterium (Table 4).

**Table 4:** 16S rDNA sequences close to full length identified in the four datasets.

Dataset	Sequence ID	Best matched 16S				Description of the next three matches^4^
			
		ID^1^	Description	Bit score^3^	Identity (%)	
*D. magna *GS 20	contig04123	S000437499	*Daphnia *endosymbiotic bacterium^2^	1970	99	uncultured *Pasteuria *sp., *P. nishizawae*, *P. penetrans*
	
	contig03555	S000446092	aquatic bacterium R1-C1	1374	98	uncultured Cytophagales bacterium, aquatic bacterium R1-C5, uncultured bacterium

*D. magna *GS FLX	contig00041	S000893806	*Shigella dysenteriae*	2627	99	*Escherichia coli *W3110, *E. coli *K12, *E. coli*
	
	contig06506	S000343002	uncultured Cytophagales bacterium	2468	96	uncultured bacterium, *Flavobacterium *sp. Nj-26, uncultured Flavobacteriales bacterium
	
	contig06300	S000372741	uncultured bacterium	1947	93	Myxococcales str. NOSO-1, *Chondromyces pediculatus*, *Polyangium thaxteri*
	
	contig06583	S000437499	*Daphnia *endosymbiotic bacterium^2^	1943	99	uncultured *Pasteuria *sp., *P. nishizawae*, *P. penetrans*

*D. pulicaria*	ANIT159445.g1	S000966592	*Flavobacterium *sp. MH45	1905	99	Arctic sea ice bacterium ARK10164, uncultured bacterium, *Flavobacterium succinicans*
	
	ANIT198306.b1	S000799101	uncultured bacterium	1857	98	Comamonadaceae bacterium BP-1b,
	
	ANIT159586.b1	S000639702	uncultured bacterium	1853	98	uncultured Burkholderiales bacterium, Comamonadaceae bacterium BP-1b, uncultured proteobacterium
	
	ANIT82605.b1	S000634984	uncultured Burkholderiales bacterium	1846	99	uncultured bacterium, Comamonadaceae bacterium BP-1b, Comamonadaceae bacterium BP-1
	
	ANIU5178.g2	S000429300	*Flavobacterium *sp. GOBB3-209	1653	98	uncultured bacterium, uncultured Cytophagales bacterium, uncultured Sphingobacteriales bacterium
	
	ANIT142825.b1	S000634984	uncultured Burkholderiales bacterium	1570	98	uncultured beta proteobacterium, uncultured organism, *Rhodoferax ferrireducens *T118
	
	ANIS174043.g1	S000446066	aquatic bacterium R1-B19	1485	99	uncultured beta proteobacterium, aquatic bacterium R1-B6, uncultured Burkholderiales bacterium
	
	ANIT169338.b1	S000005772	*Aeromonas eucrenophila*	1465	99	*Aeromonas *sp. 'CDC 859-83', *A. molluscorum*, uncultured bacterium
	
	ANIS242375.b1	S000658887	uncultured actinobacterium	1439	97	uncultured bacterium, *Modestobacter multiseptatus*, *Sporichthya polymorpha*
	
	ANIS247631.y1	S000607919	*Pseudomonas *sp. R-25061	1419	99	*Pseudomonas *sp. R-25209, uncultured bacterium, *P. pseudoalcaligenes*
	
	ANIU876.b3	S000948974	uncultured bacterium	1386	98	uncultured gamma proteobacterium, uncultured *Pseudomonas *sp., *Pseudomonas *sp. G2
	
	ANIT143068.b1	S000550675	*Pseudomonas *sp. GD100	1318	96	*Pseudomonas *sp. Pb1(2006), *P. poae*, *P. lurida*
	
	ANIT82605.g2	S000634984	uncultured Burkholderiales bacterium	1312	100	uncultured bacterium, *Variovorax paradoxus*, uncultured bacterium SJA-62
	
	ANIT131207.y2	S000018838	uncultured Cytophagales bacterium	1304	91	uncultured bacterium, uncultured Bacteroidetes bacterium, rhizosphere soil bacterium RSC-II-81
	
	ANIT102921.y2	S000895013	uncultured bacterium	1170	93	uncultured Cytophagales bacterium, uncultured Bacteroidetes bacterium, uncultured bacterium
	
	ANIU1607.g2	S000799546	uncultured bacterium	1092	96	*Hydrogenophaga *sp. AH-24, *Hydrogenophaga *sp. CL3, *Hydrogenophaga *sp. YED1-18

*D. pulex*	scaffold_278	S000541019	*Pseudomonas argentinensis*	2785	98	*P. argentinensis*, *P. fluorescens *PfO-1,
	
	scaffold_567	S000402041	uncultured bacterium	2680	97	uncultured soil bacterium, uncultured Comamonadaceae bacterium, uncultured beta proteobacterium
	
	scaffold_1523	S000926010	*Serratia proteamaculans *568	2615	96	*Serratia proteamaculans *568, uncultured bacterium, uncultured proteobacterium
	
	scaffold_6081	S000730527	*Deefgea rivuli*	1792	97	uncultured bacterium, *Chitinibacter tainanensis*, uncultured proteobacterium
	
	scaffold_16248	S000736150	gamma proteobacterium GPTSA100-21	1711	98	gamma proteobacterium GPTSA100-22, uncultured bacterium, gamma proteobacterium GPTSA100-26
	
	scaffold_10095	S000404820	*Pseudomonas *sp. Hsa.28	1378	99	uncultured bacterium, uncultured *Pseudomonas *sp., *P. anguilliseptica*
	
	scaffold_1408	S000446066	aquatic bacterium R1-B19	1326	99	uncultured beta proteobacterium, aquatic bacterium R1-B6, aquatic bacterium R1-B7
	
	scaffold_21984	S000656075	uncultured *Pseudomonas *sp.	1023	100	gamma proteobacterium LC-G-2, *Pseudomonas *sp. 7-1, *P. fluorescens*

^1 ^Given as the ID in RDP.

^2 ^*Pasteuria ramosa*, the parasite which was present in the *D. magna *datasets.

^3 ^The BLAST bit scores obtained from a comparison of the contigs/scaffolds to annotated 16S rDNA sequences present in RDP are shown. A higher number indicates a more significant match.

^4 ^The next top three unique matched species, if they were not the same as the best match

The 16S rDNA sequences identified only a small subset of the species/genus found in our main analysis based on comparison to NCBI-nt database. One likely explanation of this discrepancy is the low sequencing coverage within the 16S rDNA regions in the shotgun datasets. Another explanation could be that some of the earlier predictions were false positives. However, MEGAN associates a sequence to the lowest common ancestor of the set of taxa defined by all matches above defined thresholds. The amount of false predictions is predicted to be low since the algorithm makes higher amount of unspecific assignments to higher taxonomy levels [20]. Certainly when taxa were inferred regardless if the matched sequence was a suitable phylogenetic marker or not, it could not be excluded that some of the predictions were results of horizontal gene transfer events. However, if this were the case, MEGAN would assign the hit to the least common ancestor of the species, which were involved in horizontal gene transfer, unless neither these species nor related species are in the NCBI database. It was predicted that computing taxonomic content based on sequence comparison to NCBI-nt database will show better resolution at all levels of the taxonomy than an analysis based on a small set of phylogenetic markers or on 16S rDNA sequences alone [20,21]. Our results are consistent with this prediction.

**Figure 9:**
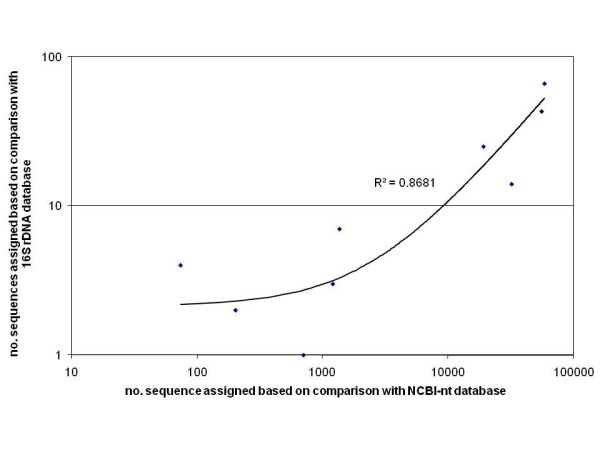
**Correlation of taxonomic content computed by comparison to NCBI-nt and comparison to 16S rDNA database**. The number of sequences assigned to the following taxonomic nodes were plotted: Bacteria, Proteobacteria, Bacteroidetes, Gammaproteobacteria, Deltaproteobacteria, Betaproteobacteria, Flavobacteria, Sphingobacteria, Actinobacteria.

Despite the under-prediction and the differences between the NCBI-nt and the 16S rDNA databases, quantitatively, the two approaches correlated fairly well at higher taxonomic level (Fig. 9).

### Searching for identical and similar sequences common in four datasets

Although sequences in all datasets were assigned to similar bacterial taxa, it is not clear how similar the sequences are across datasets. To identify common sequences, we compared the *D. magna *GS 20 sequences with sequences from *D. magna *GS FLX, *D. pulex*, and *D. pulicaria *using BLASTN. Identical or nearly identical sequences were identified when a stretch longer than 80% of a query sequence can be aligned with over 98% nucleotide sequence identity to a hit sequence. With this criterion five *D. magna *GS 20 contigs (corresponding to six *D. pulex *scaffolds and 12 *D. pulicaria *reads) were identified. Hits identical to these sequences were all found in complete genome sequences of *Escherichia coli *W3110 (AP009048.1) and *E. coli *K12 MG1655 (U00096.2), which suggests that commensal *E. coli *strains carried by the three *Daphnia *species are highly similar.

With a less stringent criterion (a stretch longer than 50% of a query sequence can be aligned with over 90% nucleotide sequence identity to a hit sequence), similar sequences to about 80 GS 20 contig sequences were also identified across the datasets. These sequences mainly fall into taxa within the Proteobacteria, with a few sequences assigned to *Flavobacterium*.

The small number of similar sequences shared across the datatsets suggested the bacterial community carried by the three *Daphnia *clones from which our datasets originated might be diverse at species and strain level, despite very high homogeneousness observed at higher taxonomy nodes. It should be noted however, that our datasets do not originate directly from field samples, but from three clones, which had been kept in three different laboratories for several generations before the DNA was isolated. This may possibly influence our results in two ways. First, we cannot truly make statements about three *Daphnia *species, but only about three clones, each coming from a different *Daphnia *species. Including more clones, might reveal more bacterial symbionts. Second, while culturing these clones in the laboratory, the symbiont community may have changed both qualitatively and quantitatively. New bacterial species may have arrived with food or culture conditions, while other bacteria may have been lost due to the inappropriateness of the laboratory conditions for their culture. For the current analysis, no attempts have been undertaken to vary the culture conditions for any of the three clones and the bacteria associated with the food alga have not been analyzed.

### Repeatability of the metagenomics approach

**Figure 10:**
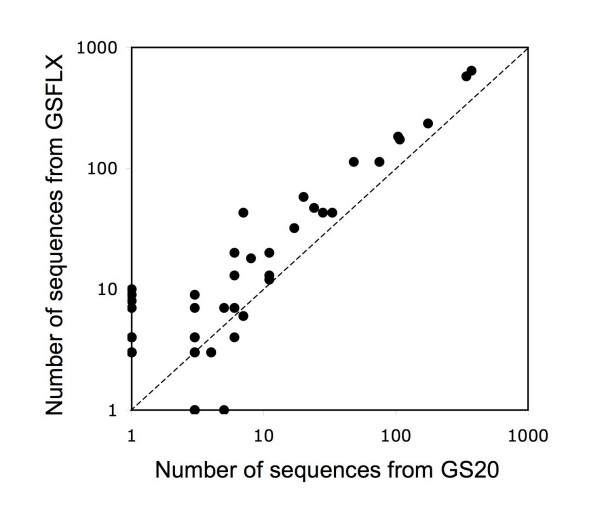
**Comparison of the number of assigned sequences (log_10_(x+1)) to prokaryote genera (excluding Firmicutes) of the combined two *D*. magna datasets**.

For *D. magna *we obtained two shotgun datasets, with sequences produced with two different sequencing platforms, the pyrosequencers GS 20 and GS FLX. Figure 10 shows the number of sequences assigned to all prokaryote genera (excluding the Firmicutes) in the two datasets. The two datasets gave very congruent results, with a correlation coefficient of *r *= 0.98 (P < 0.001, n = 55). The plot shows clearly that stochastic differences occur for genera with very few hits. Expectedly, below 10 sequences assigned to a genus, the datasets lead to quite divergent result.

### Using contigs instead of reads

For the *D. pulicaria *dataset, both contigs and singleton raw reads were included in our analysis. For the other three datasets, we used only sequences, which had previously been assembled to contigs or scaffolds. This reduced the number of sequences and thus the number of BLASTN searches considerably. Using large numbers of raw reads would have been beyond our computing power and the abilities of the MEGAN software within a reasonable time period. Using contigs and scaffolds influences the results in various ways. First, it strongly reduces redundancy in the dataset and therefore makes the analysis much quicker. Second, it compromises somewhat the usefulness of the number of assigned sequences as a measure for the abundance for the different taxa. The number of assigned sequences is still a relative measure for the frequency of a given taxa, but the larger the real number of hits would have been, the more strongly the value is reduced. Third, rare members of the symbiont community are likely to remain undetected, because the few reads sequenced for rare species, were unlikely to be assembled in contigs. Thus, our estimates of the number of taxa detected are likely to underestimate the true number of taxa in the community. This conclusion is also supported by the observation that the *D. pulicaria *dataset contained the highest number of taxa identified.

## Conclusion

Our analysis of shotgun sequences of three clones, each from one *Daphnia *species revealed a rich bacterial community to be associated with these clones. The particular data structure of our analysis allows for certain conclusions to be drawn. First, the majority of the common bacterial taxa identified are found in all *Daphnia *datasets. While the *D. pulex *and *D. pulicaria *clone cultures from which DNA was isolated originated from laboratories in North America, the *D. magna *cultures originate from a laboratory in Switzerland. To the best of our knowledge, there was never a cross Atlantic exchange of cultures between laboratories by the time these samples had been taken. Thus, we speculate that the similarity of the symbiont communities in European and North American *Daphnia *samples, indicates a long lasting stability of these associations.

Second, the symbiont communities across the three *Daphnia *species are remarkable similar, yet, they are not identical. At sequence level, the similarity breaks down, indicating that each *Daphnia *species harbors different species or strains of bacterial symbionts.

Third, some bacterial taxa were found to be specific to the two datasets produced in the DOE Joint Genome Institute (JGI). Coincidentally, some of the published genomes in these taxa had been originally sequenced by JGI, leading to speculations of whether the JGI may have contaminated the *Daphnia *samples. Our analysis allows us clearly to reject this hypothesis. Whether the bacterial taxa found to be associated with specific *Daphnia *samples are contaminations of the laboratory where they were cultured previous to sequencing, or if they are natural symbionts of the *Daphnia*, cannot not be worked out here.

Fourth, there is no clear evidence for a stable cyanobacterial or plastid symbiont in the *Daphnia *species. The few scattered hits to some plastid and Cyanobacteria may have been a contamination with the algae food of the *Daphnia*. Plastid symbionts had been observed in *D. obtusa *[37]. However, the long laboratory culture of the clones used in the genome study may have influenced the presence of such a photoactive symbiont.

## Methods

### The *D. pulex *dataset

The sequences of *D. pulex *are from the DGC whole genome sequencing project. The chosen *D. pulex *clone called The Chosen One was cultured at Indiana University, Bloomington, USA on a diet of the green algae *Scenedesmus *sp. The animals used to isolate the DNA for the genome project were treated with tetracycline (250 mg/L overnight) before DNA isolation to reduce their bacterial load. Sequencing was done at the DOE Joint Genome Institute (JGI) using the Sanger method. These sequences were obtained from the wFleaBase website . Scaffolds included in this study were excluded scaffolds, prokaryotic scaffolds, and possible bacterial scaffolds in the current *D. pulex *genome assembly .

### The *D. pulicaria *dataset

*Daphnia pulicaria *is closely related to *D. pulex *and forms with intermediate characters are frequently encountered, suggesting hybridization of these two species. Indeed, allozyme test for allelic variation at the lactate dehydrogenase loci show both fast and slow electromorphic alleles, indicating that the chosen *D. pulicaria *strain is a pulicaria/pulex hybrid. This chosen *D. pulicaria *clone was cultured at the Hubbard Center for Genome Studies at the University of New Hampshire, USA, on a diet of the green algae *Ankistrodesmus falcatus*. Previous to it's culturing at the University of New Hampshire it was maintained in a laboratory at Utah State University. The animals used to isolate the DNA for the genome project were treated with tetracycline (250 mg/L overnight) before DNA isolation to reduce their bacterial load. Sequencing of *D. pulicaria *was also done at the DOE Joint Genome Institute (JGI) using the Sanger method. A low coverage genome assembly of a *D. pulicaria *clone is available to DGC members, and others may request access to this data. As the DGC and JGI data agreements allow, this will be released for public access on the wfleabase database: . For more information on the *D. pulex *and *D. pulicaria *genome data see .

### The *D. magna *datasets

The sequences of *D. magna *originated from a shotgun sequencing project which aimed at sequencing the endoparasitic bacterium *P. ramosa*. During the analysis of the data large number of sequences clearly unrelated to the Firmicutes (the group to which *P. ramosa *belongs) showed up. Only these sequences are included in this paper. As these data are not yet published elsewhere, we describe here the DNA isolation, library construction and sequencing in detail.

*Daphnia magna *cultures were raised at the University of Fribourg, Switzerland on a diet of the green algae *Scenedesmus *sp. The *Daphnia *had been exposed to the gram-positive bacterium *Pasteuria ramosa*, an endo-parasite of *Daphnia *[17] when they were 3–5 days old. Most animals became infected and were shipped for further processing to the University of Florida, USA. One thousand *P. ramosa *infected *D. magna *were suspended in 5 ml of Buffer A (1.0 M NaCl, 50 mM Tris-HCl pH 8.0) and homogenized gently in a glass pestle and mortar. The homogenate was passed through a 50–100 micron metal mesh and 21 micron nylon mesh to remove *Daphnia *debris. About 5,000,000 *P. ramosa *cells were obtained and resuspended in 450 μl of Buffer A. These were added to an equal volume (450 μl) of 2% agarose for preparing a gel plug to embed the vegetative cells, and 10 gel plugs were produced. To disrupt cells gently, the gel plugs were transferred into Buffer B (0.2% sodium deoxycholate, 0.5% Brij 58, 0.5% sarcosine, 50 mM Tris-HCl pH 8.0, 100 mM EDTA pH 8.0, 0.40 M NaCl) and incubated at 37°C overnight. These were then transferred into 10 ml of Buffer C (100 mM NaCl, 50 mM Tris-HCl pH 8.0, 100 mM EDTA pH 8.0, 0.5% sarcosine, 0.2 mg/ml protease K) at room temperature. The gel plugs were transferred to 40 ml of Wash Buffer (10 mM Tris-HCl pH 8.0, 10 mM EDTA pH 8.0) and washed three times in a shaker at low speed for 1 hourrespectively to remove detergents. Gel plugs were transferred to 40 ml of PMSF Buffer (1.0 mM phenylmethylsulfonyl floride PMSF, 10 mM Tris-HCl pH 8.0, 10 mM EDTA pH 8.0) and incubated at room temperature for 1 hourwith gentle shaking; this process was repeated with fresh PMSF buffer. The plugs were then washed twice in 40 ml of Wash Buffer following incubation at 50°C for 20 minutes. The gel plugs were then transferred to 40 ml of 50 mM EDTA (pH 8.0) and stored at 4°C overnight. The DNA in the gel plugs was digested with 10 U of HindIII per plug at 37°C for 30 minutes.

The gel plugs with the partially digested DNA were cut into slurry. They were loaded onto a 1% agraose gel (Sigma, Type VII, low gelling temperature), and sealed on the top with agarose. Electrophoretic development occurred in 0.7 × TAE Buffer using a FIGE apparatus under Program 4 (BioRad, Hercules, CA 94547). Products ranging in size from 18 to 33 Kb were extracted from the gel (estimated 60 ng DNA total) following the protocol of GELase Agarose Gel-Digesting Preparation kit (Epicentre, Madison, WI 53713), and used to prepare the cosmid library.

The preparation of the cosmid library followed the procedures described by Bell et al. [38], with additional information described by Chow et al. [39]. In brief to construct the cosmid library an estimated 60 ng of 18–33 Kb fragments recovered from gel were cloned into vector pCC1 which was digested with HindIII and then dephosphorylated with shrimp alkaline phosphatase followed the protocol (Roche, Indianapolis, IN 46250). The ligation products were packaged into bacteriophage particles using MaxPlax Lamda DNA packaging extracts (Epicentre, Madison, WI 53713) according to the protocol of the kit. Bacteriophage containing an estimated 5 × 10^3 ^particles in 50 μL were applied to infect 200 μl of EPI300 cells grown to exponential phase in LB liquid medium (Luria-Bertani medium) containing 10 mM MgSO4 and 0.2% maltose, which had been inoculated from the overnight culture grown in LB containing 10 mM MgSO_4_. After absorption following incubating at 37°C for 20 minutes, 1 ml of fresh LB medium was added and incubated for an additional 45 minutes. The infected cells were spread on LB 1% agar plates containing 12.5 μg/ml of chloramphenicol, 1 mM of IPTG and 40 μg/ml of X-gal for selection.

The cosmid library was used in two runs of 454 pyrosequencing [26]. The first run was carried out on a GS 20 454 pyrosequencer, which gave read length around 90 basepairs (bps). The second run was done on a GS FLX 454 pyrosequencer, which gave reads length around 250 bps. Both pyrosequencing projects were done in the Interdisciplinary Center for Biotechnology Research at the University of Florida, Gainesville, USA. The reads obtained from the GS 20 and the GS FLX shotgun sequencing were separately assembled into contigs. These contigs were used in the analyses presented here.

### Scanning electron microscopy

For scanning electron microscopic (SEM) *D. magna *was fixed in 3% glutaraldehyde in 0.1 M PB for 2 hours at 20°C. Sample was washed two times in distilled water for 5 to 10 seconds, dehydrated in graded ethanol series, and critical point dried (CPD) overnight (16 hours). The specimens were coated with gold (20 nm) and viewed using a Philips XL 30 ESEM under high volume conditions from 5 to 15 kv.

### Data analysis

Sequences from the *D. pulex*, *D. pulicaria *and the two *D. magna *datasets included in this study are described in Table 2. Sequences were compared against the NCBI-nt database on nucleotide sequences using BLASTN [19] with the default settings in December 2007. Sequences longer than 1000 bps were divided into overlapping fragments around 500 bps. Sequences were homogenized to fragments of similar length so BLAST scores were comparable across different searches. Sequence comparison is computational challenging and was performed with an Opteron Linux high performance computer cluster established and maintained by the [BC]^2 ^Basel Computational Biology Center at the Biozentrum University of Basel . For the graphical presentation of the results we combined the two *D. magna *data sets.

For the analysis of the BLASTN results we used the metagenomics software MEGAN [20]. This software allows exploring the taxonomic content of a sample based on the NCBI taxonomy. The blast files were imported into MEGAN using the import option BLASTN. The program then uses several thresholds to generate sequence-taxon matches. The "min-score" filter sets a bit-score cutoff value. The "top-percent" filter is used to retain hits whose scores lie within a given percentage of the highest bit score. The "min-support" filter is used to set a threshold for the minimum number of sequences that must be assigned to a taxon. We used all default parameter settings of the software (top-percent = 10, min-support = 2), except the minimal threshold for the bit score of hits, which were set at 100, following the recommendation of the authors [20]. This reduces the number of reads assigned to a taxon, but avoids assignment based on weak homology. This analysis was done for all datasets between the 8. and the 11. January 2008.

While inspecting the data we ignored reads assigned to taxa other than plants and bacteria. Within the bacteria, we ignored the taxon Firmicutes (mostly gram-positive bacteria, many of which are endospore formers), because the two datasets of *D. magna *came from animals infected with the endospore forming pathogen, *P. ramosa*. The two other datasets (*D. pulex *and *D. pulicaria*), had only few sequences assigned to the Firmicutes (less than 0.2%). Thus, excluding the Firmicutes from the analysis did not influence the overall analysis.

In a separate analysis we manually inspected all four datasets for hits assigned to plant taxa (every taxon within and including the Viridiplantae), searching for hits to plastids (chloroplasts). For this analysis we set the MEGAN parameter minimum supported taxa to one.

## Authors' contributions

WQ and DE carried out the Bioinformatics analysis. NG and JP produced the *D. magna *sequences. DE designed the study. FBA produced the SEM images. DE and WQ wrote most of the manuscript. All authors took part in reviewing and approval of the final manuscript.
